# Designing of multi-epitope chimeric vaccine using immunoinformatic platform by targeting oncogenic strain HPV 16 and 18 against cervical cancer

**DOI:** 10.1038/s41598-022-13442-4

**Published:** 2022-06-09

**Authors:** Anoop Kumar, Utkarsha Sahu, Pratima Kumari, Anshuman Dixit, Prashant Khare

**Affiliations:** 1 National Institute of Biologicals (NIB), Noida, Uttar Pradesh India; 2 Department of Microbiology, All India Institute of Medical Sciences, Bhopal, Madhya Pradesh 462020 India; 3 Institute of Life Science, Nalco Square, Bhubaneswar, Odisha 751023 India; 4 Division of Synthetic Biology, Absolute foods, 5th floor, Plot 68, Sector 44, Gurugram, Haryana 122003 India; 5 Regional Centre for Biotechnology (RCB), 3rd Milestone, Faridabad-Gurugram Expressway, Faridabad Rd, Faridabad, Haryana 121001 India

**Keywords:** Cancer, Immunology

## Abstract

Cervical cancer is the most common gynaecological cancer and reaches an alarming stage. HPVs are considered the main causative agents for cervical cancer and other sexually transmitted infections across the globe. Currently, three prophylactic vaccines are available against HPV infections with no therapeutic values. Due to a lack of effective therapeutic and prophylactic measures, the HPV infection is spreading in an uncontrolled manner. Next-generation of vaccine is needed to have both prophylactic and therapeutic values against HPV. Here first time we have designed a multi-epitope chimeric vaccine using the most oncogenic strain HPV 16 and HPV 18 through an immunoinformatic approach. In this study, we have used the L1, E5, E6 and E7 oncoproteins from both HPV 16 and HPV 18 strains for epitope prediction. Our recombinant chimeric vaccine construct consists, selected helper and cytotoxic T cell epitopes. Our computational analysis suggests that this chimeric construct is highly stable, non-toxic and also capable of inducing both cell-mediated and humoral immune responses. Furthermore, in silico cloning of the multi-epitope chimeric vaccine construct was done and the stabilization of the vaccine construct is validated with molecular dynamics simulation studies. Finally, our results indicated that our construct could be used for an effective prophylactic and therapeutic vaccine against HPV.

## Introduction

Human papillomaviruses (HPVs) are ubiquitous double-stranded DNA (dsDNA) viruses belonging to family *papillomaviridae*. HPV induces hyperplastic, papillomatous, and verrucous squamous cell lesions in the skin and at various mucosal sites in a wide range of hosts, including humans and exploits the cellular machinery for their own purposes. Of more than 200 genotypes that have been described till date^1^, some are categorized as high-risk (HR-HPVs) types (HPV 16, 18, 31, 33. 35, 39, 45, 51, 52, 56, 58, 59, 68, 73 and 82) which are associated with genital and other epithelial cancers, and others as low-risk (LR-HPVs) types (HPV6, 11, 40, 42, 43, 44, 54, 61, 70, 72, 81, CP6108), which are responsible for benign tumors and genital warts^2–4^. Worldwide, it is one of the most common sexually transmitted infections, causing approximately 5% of all cancer cases^5,6^. Out of 200 HPVs, mainly HPV 16 and HPV 18, are the crucial etiological agents for several epithelial cell malignancies.

HPVs are small, non-enveloped, circular dsDNA viruses with ~ 8 kb genome containing three regions, late proteins (L1 and L2), early proteins (E1, E2, E4, E5, E6, and E7) and upstream regulatory region (URR). Out of 6 early proteins, E5, E6, and E7 play a crucial role in the transformation of cell. Two early oncoproteins, E6 and E7, are constantly expressed in almost all cervical cancer cells and identified as ideal targets for the immunotherapy of HPV-associated cancers^7,8^.

The World Health Organization (WHO) prequalified three vaccines against HPV i.e., a bivalent, a quadrivalent and nonavalent vaccine are being administered to encounter HPV 16 & HPV 18 infection which are responsible for approximately 70% of global cervical cancer cases (https://www.who.int/teams/immunization-vaccines-and-biologicals/diseases/human-papillomavirus-vaccines-(HPV). The quadrivalent vaccine having an additional impact on LR-HPV 6 & HPV 11 and showed efficacy in preventing anogenital warts caused by HPV 6 & 11 (https://www.who.int/immunization/hpv/vaccines/en/). However, none of them have therapeutic effects^9,10^. Due to the high prevalence and mortality with HPV associated cervical cancer, an effective therapeutic HPV vaccine or a vaccine having both prophylactic and therapeutic properties is urgently required for the clearance of the virus from the host.

Peptide-based vaccines have certain advantages as they are easy to produce and transport, have high selectivity, multivalency competence and have accessibility to the epitope. With the advancement in genome sequencing techniques, potential B and T cell epitopes can be predicted and have a promising outlook for the development of the peptide-based vaccine against any infectious agent or disease and/or cancers. At present, a branch of bioinformatics, immunoinformatics, used for the identification of effective T- & B- cell immunogenic epitopes from the antigenic peptide by different software which aids us to classify cost and labor effective immunogenic peptides by eluding the non-immunogenic sequence. These methods aid as low-cost by saving the cost of synthetic peptide expense of and working time^11,12^.

**Figure 1:**
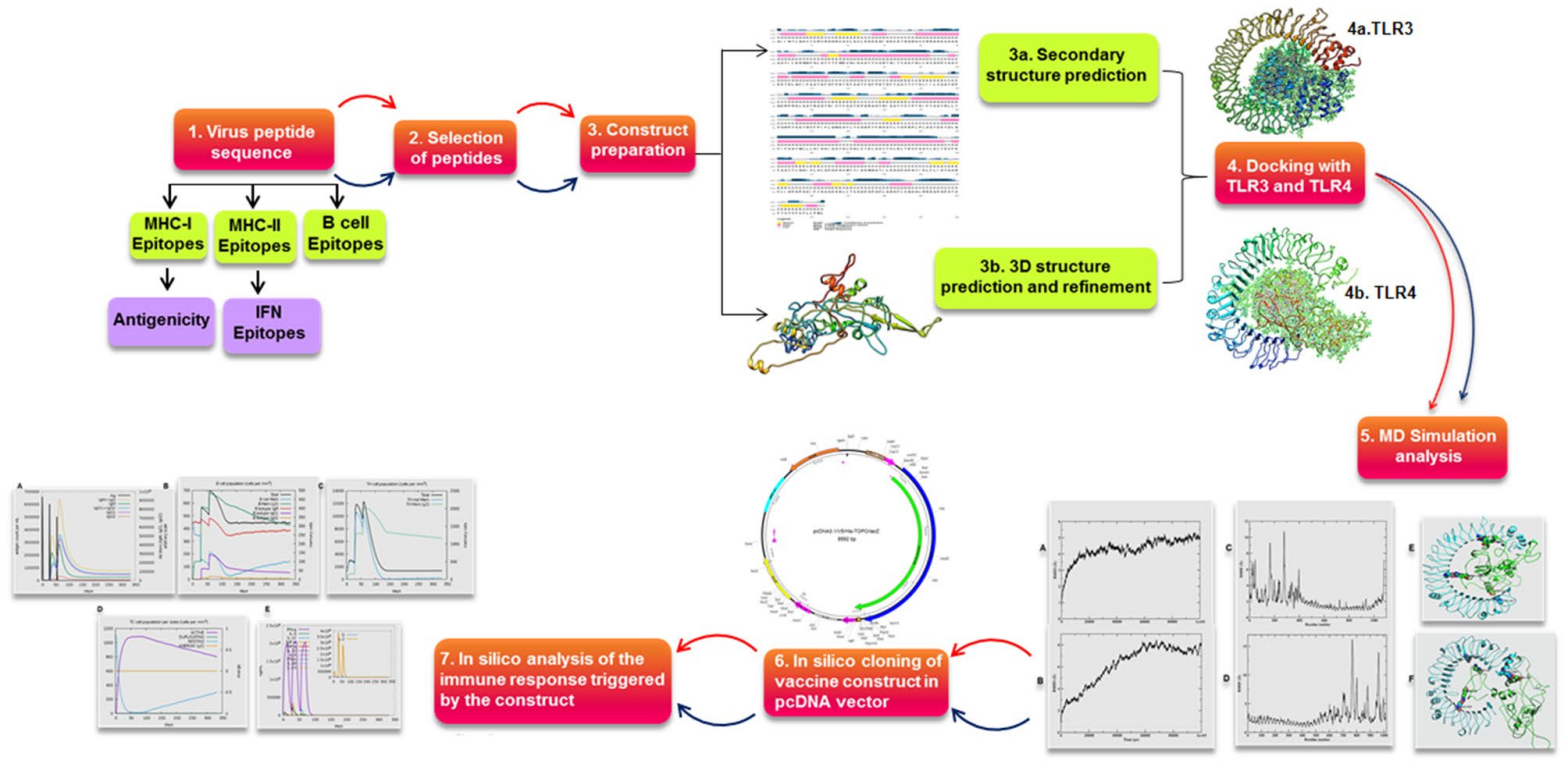
An overview of steps used in designing the chimeric vaccine construct.

The polymorphism in host genetics can impact the immune response to a pathogen in the target population. In view of the polymorphic nature of major histocompatibility complex (MHC) class I and II alleles they bind towards specific repertoire, short peptides (epitopes), from the processed pathogen and presentation of these complexes to T cells is very important for the development of effective cytotoxic T lymphocytes (CTL) response against the pathogen^13^. The present study focused on the development of the effective chimeric vaccine having both prophylactic and therapeutic effects against the two HR-HPV (HPV 16 & 18) by reverse vaccinology approach. Here we have analyzed the L1, E5, E6 and E7 proteins of both HPV 16 and 18 by employing various algorithms for the prediction of the best T-cell and B-cell epitopes. Next, we have constructed a multi-epitope chimeric construct and evaluated all physiochemical parameters along with its antigenicity and allergenicity. Furthermore, we have done in silico cloning and checked the stability of our construct through MD simulation. The complete design and methods were used in this study are depicted in Fig. 1.

## Results

### T lymphocyte epitopes

Since epitopes on T cells are bound to MHCs, hence their interaction can be modeled accurately on the basis of certain algorithms. Both MHC-I & MHC-II epitopes are needed to make an immunogenic vaccine construct. Here, we have predicted the potent MHC-I & MHC-II epitopes using IEDB server on the default parameters.

#### MHC-I epitopes prediction

For MHC-I, the prediction of the epitopes of L1, E5, E6 & E7 proteins of HPV 16 & 18 has been done for the 27 reference alleles. The epitopes having a percentile score less than 0.5 have been short listed. The epitopes having the antigenicity score greater than 0.4 were considered as probable antigens.

##### HPV 16

A total of 155, 12, 22 and 17 epitopes having the percentile score < 0.5 in L1, E5, E6 & E7 proteins of HPV 16, respectively, has been selected for further analysis. These predicted epitopes were further analyzed for the antigenicity score and MHC-I immunogenicity. On analysis, 18, 03, 05 & 04 were found to have antigenicity score 0.4 and predicted as probable antigen for L1, E5, E6, & E7 protein, respectively (Table 1).

**Table 1:** MHC-I epitopes of L1, E5, E6, & E7 proteins of HPV 16.

S. No	Start	Peptide	Allele	Percentile_ Rank	MHC-1 immunogenicity	Antigenicity
**L1**						
1.	443	KYTFWEVNL	HLA-A*24:02; HLA-A*23:01	0.12	0.47141	1.1010 (Probable ANTIGEN)
2.	444	**YTFWEVNLK**	HLA-A*68:01; HLA-A*11:01	0.06	0.38531	1.3959 (Probable ANTIGEN)
3.	398	**ILEDWNFGL**	HLA-A*02:01; HLA-A*02:06	0.09	0.38303	1.5794 (Probable ANTIGEN)
4.	244	DSLFFYLRR	HLA-A*33:01	0.07	0.2456	0.7108 (Probable ANTIGEN)
5.	186	PPLELINTV	HLA-B*51:01	0.1	0.22885	0.7688 (Probable ANTIGEN)
6.	27	**YVARTNIYY**	HLA-A*26:01; HLA-A*01:01; HLA-A*30:02; HLA-B*35:01; HLA-B*15:01	0.04	0.20665	0.4938 (Probable ANTIGEN)
7.	45	VGHPYFPIK	HLA-A*30:01	0.14	0.17434	1.0927 (Probable ANTIGEN)
8.	330	FVTVVDTTR	HLA-A*68:01	0.19	0.17066	0.9193 (Probable ANTIGEN)
9.	41	**RLLAVGHPY**	HLA-A*32:01; HLA-B*15:01; HLA-A*30:02	0.06	0.12869	0.7359 (Probable ANTIGEN)
10.	133	SAYAANAGV	HLA-A*68:02	0.17	0.123	0.6532 (Probable ANTIGEN)
11.	42	LLAVGHPYF	HLA-B*15:01	0.24	0.10617	0.7709 (Probable ANTIGEN)
12.	247	FFYLRREQM	HLA-B*08:01	0.05	0.10358	1.1625 (Probable ANTIGEN)
13.	368	**EEYDLQFIF**	HLA-B*44:03; HLA-B*44:02; HLA-B*40:01	0.01	0.07784	1.7384 (Probable ANTIGEN)
14.	82	**KFGFPDTSF**	HLA-A*23:01; HLA-A*24:02	0.11	0.07498	1.5682 (Probable ANTIGEN)
15.	422	TSQAIACQK	HLA-A*11:01	0.23	0.05502	0.7828 (Probable ANTIGEN)
16.	105	VEVGRGQPL	HLA-B*40:01	0.1	0.02556	1.2418 (Probable ANTIGEN)
17.	83	**FGFPDTSFY**	HLA-B*35:01; HLA-A*30:02; HLA-A*01:01	0.08	0.01376	1.0287 (Probable ANTIGEN)
18.	348	ISTSETTYK	HLA-A*11:01	0.23	0.01077	0.7773 (Probable ANTIGEN)
**E5**						
19.	71	LFLIHTHAR	HLA-A*33:01	0.11	0.24852	0.5205 (Probable ANTIGEN)
20.	72	FLIHTHARF	HLA-B*15:01	0.2	0.20726	0.6381 (Probable ANTIGEN)
21.	38	**TYTSLIILV**	HLA-A*24:02; HLA-A*23:01	0.18	0.06645	0.6505 (Probable ANTIGEN)
**E6**						
22.	134	NIRGRWTGR	HLA-A*33:01	0.13	0.36237	2.1770 (Probable ANTIGEN)
23.	53	AFRDLCIVY	HLA-A*30:02	0.17	0.11401	1.8618 (Probable ANTIGEN)
24.	80	ISEYRHYCY	HLA-A*01:01	0.07	0.07501	1.4506 (Probable ANTIGEN)
25.	11	**DPQERPRKL**	HLA-B*08:01; HLA-B*51:01	0.05	0.02079	0.5782 (Probable ANTIGEN)
26.	87	CYSLYGTTL	HLA-A*24:02	0.23	0.01888	0.4301 (Probable ANTIGEN)
**E7**						
27.	15	LQPETTDLY	HLA-B*15:01	0.14	0.18373	1.1847 (Probable ANTIGEN)
28.	49	**RAHYNIVTF**	HLA-A*32:01; HLA-B*15:01; HLA-B*58:01; HLA-B*57:01; HLA-B*35:01	0.04	0.18328	0.5919 (Probable ANTIGEN)
29.	46	**EPDRAHYNI**	HLA-B*51:01; HLA-B*53:01	0.23	0.12093	0.6109 (Probable ANTIGEN)
30.	82	LLMGTLGIV	HLA-A*02:03	0.22	0.11082	1.2628 (Probable ANTIGEN)

Bold text represents the peptides used in vaccine construct.

##### HPV 18

A total of 155, 13, 38 and 9 epitopes having the percentile score < 0.5 in L1, E5, E6 & E7 proteins of HPV 18, respectively, has been selected for further analysis. These predicted epitopes were further analyzed for the antigenicity score and MHC-I immunogenicity. The epitopes having an antigenicity score greater than 0.4 and probable antigen. On analysis, 19, 09, 02 & 01 were found to have antigenicity score 0.4 and predicted as probable antigen for L1, E5, E6, & E7 protein, respectively (Table 2).

**Table 2:** MHC-I epitopes of L1, E5, E6, & E7 proteins of HPV 18.

S. No	Start	Peptide	Allele	Percentile rank	MHC-1 immunogenicity	Antigenicity
**L1**						
1.	473	TTSLVDTYR	HLA-A*68:01, HLA-A*33:01,	0.02	0.0268	0.4094 (Probable ANTIGEN)
2.	88	**YVTPTSIFY**	HLA-B*53:01, HLA-B*35:01, HLA-B*15:01, HLA-A*30:02, HLA-A*26:01, HLA-A*01:01,	0.16	0.0642	0.5066 (Probable ANTIGEN)
3.	7	VLILHYHLL	HLA-B*08:01,	0.14	0.0809	0.5274 (Probable ANTIGEN)
4.	102	**RLLTVGNPY**	HLA-B*15:01, HLA-A*32:01, HLA-A*30:02,	0.11	0.0956	0.5385 (Probable ANTIGEN)
5.	45	YIILFLRNV	HLA-A*02:06, HLA-A*02:03,	0.2	0.1755	0.6869 (Probable ANTIGEN)
6.	19	GPLYHPRPL	HLA-B*07:02,	0.04	0.0509	0.7965 (Probable ANTIGEN)
7.	54	**NVFPIFLQM**	HLA-B*35:01, HLA-A*68:02, HLA-A*32:01, HLA-A*26:01, HLA-A*02:06,	0.22	0.1896	0.8028 (Probable ANTIGEN)
8.	305	DSMFFCLRR	HLA-A*33:01,	0.06	0.1449	0.8574 (Probable ANTIGEN)
9.	105	TVGNPYFRV	HLA-A*68:02,	0.13	0.1193	0.8608 (Probable ANTIGEN)
10.	335	SLYIKGTGM	HLA-B*08:01,	0.16	0.0072	0.8897 (Probable ANTIGEN)
11.	47	ILFLRNVNV	HLA-A*02:03, HLA-A*02:01,	0.06	0.1022	1.0596 (Probable ANTIGEN)
12.	9	ILHYHLLPL	HLA-A*02:03,	0.25	0.0120	1.1935 (Probable ANTIGEN)
13.	104	**LTVGNPYFR**	HLA-A*68:01, HLA-A*33:01, HLA-A*31:01,	0.05	0.0960	1.2652 (Probable ANTIGEN)
14.	166	VEIGRGQPL	HLA-B*40:01,	0.07	0.0554	1.2796 (Probable ANTIGEN)
15.	428	HVEEYDLQF	HLA-B*35:01,	0.24	0.0735	1.4499 (Probable ANTIGEN)
16.	21	**LYHPRPLPL**	HLA-B*08:01, HLA-A*24:02, HLA-A*23:01,	0.09	0.0235	1.5674 (Probable ANTIGEN)
17.	484	QSVAITCQK	HLA-A*11:01,	0.25	0.1057	1.5950 (Probable ANTIGEN)
18.	430	**EEYDLQFIF**	HLA-B*44:03, HLA-B*44:02, HLA-B*40:01,	0.01	0.0778	1.7384 (Probable ANTIGEN)
19.	308	FFCLRREQL	HLA-B*08:01,	0.22	0.0873	1.8410 (Probable ANTIGEN)
**E5**						
20.	61	**MLLLHIHAI**	HLA-A*02:01, HLA-A*02:03, HLA-A*02:06, HLA-B*08:01,	0.12	0.19218	0.4891 (Probable ANTIGEN)
21.	31	YAWVLVFVY	HLA-B*35:01	0.13	0.26442	0.4484 (Probable ANTIGEN)
E6						
22.	13	**KLPDLCTEL**	HLA-A*02:01, HLA-A*02:03, HLA-A*02:06,	0.1	0.04843	0.9489 (Probable ANTIGEN)
23.	36	KTVLELTEV	HLA-A*02:06,	0.1	0.18056	0.9851 (Probable ANTIGEN)
24.	37	**TVLELTEVF**	HLA-B*35:01, HLA-B*53:01,	0.13	0.23151	0.7271 (Probable ANTIGEN)
25.	39	**LELTEVFEF**	HLA-B*44:03, HLA-B*44:02,	0.17	0.32912	1.0208 (Probable ANTIGEN)
26.	40	ELTEVFEFA	HLA-A*68:02,	0.17	0.41665	0.4910 (Probable ANTIGEN)
27.	45	FEFAFKDLF	HLA-B*40:01, HLA-B*44:03,	0.15	0.00061	1.6451 (Probable ANTIGEN)
28.	70	DFYSRIREL	HLA-B*08:01,	0.05	0.11019	0.5919 (Probable ANTIGEN)
29.	90	LEKLTNTGL	HLA-B*40:01,	0.15	0.00311	0.8025 (Probable ANTIGEN)
30.	126	RFHNIAGHY	HLA-A*30:02,	0.03	0.21792	0.5004 (Probable ANTIGEN)
**E7**						
31.	7	**TLQDIVLHL**	HLA-A*02:01, HLA-A*02:06, HLA-A*02:03	0.01	0.16272	0.5149 (Probable ANTIGEN)

Bold text represents the peptides used in vaccine construct.

#### MHC-II epitopes prediction

For MHC-II, the prediction of the epitopes of L1, E5, E6 & E7 proteins of HPV 16 & 18 has been made for the 7 allele HLA reference set. The epitopes having a percentile score less than 0.5 are short-listed. A total of 22 and 24 epitopes of the L1 & E5 protein of HPV 16 were predicted for the 7 allele HLA reference set however, no predication has been made for the E6 & E7 protein of the HPV 16. For HPV 18, L1, E5, E6 & E7 protein showed the 36, 27, 06, 04 epitopes having the percentile score less than 0.5. These predicted epitopes of HPV 16 & HPV 18 were further analyzed for the interferon- γ (IFN-γ) score (positive IFN-γ Score). In HPV 16, out of 22 and 24 epitope of the L1 & E5 protein, only 09, & 03 epitopes had the positive IFN-γ Score (Table 3). For HPV 18, out of 36, 27, 06, 04 epitopes, only 07, & 03 epitopes of L1 & E5 protein and none for E6 & E7 protein had the positive IFN-γ Score (Table 4).

**Table 3:** MHC-II epitopes of L1 and E5 proteins of HPV 16.

Allele	Start	End	Length	Peptide	Percentile rank	IFNepitope	
**L1**							
HLA-DRB1*09:01	30	44	15	RTNIYYHAGTSRLLA	0.18	POSITIVE	0.080840054
HLA-DRB1*04:05	387	401	15	**VMTYIHSMNSTILED**	0.32	POSITIVE	1
HLA-DRB1*09:01	386	400	15	DVMTYIHSMNSTILE	0.4	POSITIVE	1
HLA-DRB1*09:01	385	399	15	ADVMTYIHSMNSTIL	0.42	POSITIVE	1
HLA-DRB1*04:05	386	400	15	DVMTYIHSMNSTILE	0.43	POSITIVE	1
HLA-DRB1*09:01	387	401	15	VMTYIHSMNSTILED	0.46	POSITIVE	1
HLA-DRB3*02:02	386	400	15	DVMTYIHSMNSTILE	0.49	POSITIVE	1
**E5**							
HLA-DRB1*07:01	69	83	15	IPLFLIHTHARFLIT	0.03	POSITIVE	0.26634664
HLA-DRB1*07:01	68	82	15	**YIPLFLIHTHARFLI**	0.03	POSITIVE	0.35385852
HLA-DRB1*07:01	67	81	15	VYIPLFLIHTHARFL	0.06	POSITIVE	0.21610822

Bold text represents the peptides used in vaccine construct.

**Table 4:** MHC-II epitopes of L1 & E5 proteins of HPV 18.

Allele	Start	End	Length	Peptide	Percentile_Rank	IFNepitope	
**L1**							
HLA-DRB1*09:01	93	107	15	**SIFYHAGSSRLLTVG**	0.07	POSITIVE	0.016075751
HLA-DRB5*01:01	527	541	15	GRKFLVQAGLRRKPT	0.13	POSITIVE	0.14099948
HLA-DRB5*01:01	526	540	15	LGRKFLVQAGLRRKP	0.13	POSITIVE	0.18360012
HLA-DRB5*01:01	525	539	15	**PLGRKFLVQAGLRRK**	0.13	POSITIVE	0.2995766
HLA-DRB5*01:01	528	542	15	RKFLVQAGLRRKPTI	0.13	POSITIVE	0.075489966
HLA-DRB5*01:01	524	538	15	YPLGRKFLVQAGLRR	0.13	POSITIVE	0.067220048
HLA-DRB1*07:01	93	107	15	SIFYHAGSSRLLTVG	0.35	POSITIVE	0.016075751
**E5**							
HLA-DPA1*01:03/DPB1*02:01	47	61	15	ATAFTVYVFCFLLPM	0.37	POSITIVE	0.37550945
HLA-DPA1*01:03/DPB1*02:01	48	62	15	**TAFTVYVFCFLLPML**	0.37	POSITIVE	0.41386704

Bold text represents the peptides used in vaccine construct.

### Linear B cell epitopes

The B cell epitopes for L1, E5, E6 & E7 proteins of HPV 16 & 18 were predicted by using the ABCpred server (Tables 5 and 6). The final construct was subjected to ABCpred for the prediction of the potential B cell epitope at a cut-off of 0.8 and 24 potential epitopes were predicted in the multi-epitope chimeric vaccine construct (Table 7). The most potential B cell epitopes were LQFIFAAYKFGFPDTS, PGSIFYHAGSSRLLTV, & PGTAFTVYVFCFLLPM having score more than 0.90, 0.89 & 0.88, respectively. The B cell epitopes are important in the antigenic determinants and recognized by receptors on B lymphocytes.

**Table 5:** B Cell epitopes of L1, E6, & E7 proteins of HPV 16.

Rank	Sequence	Start position	Score
**L1**			
1	CTSICKYPDYIKMVSE	225	0.95
2	VSKVVSTDEYVARTNI	18	0.93
3	RIHLPDPNKFGFPDTS	74	0.92
3	ASSNYFPTPSGSMVTS	287	0.92
4	EATVYLPPVPVSKVVS	8	0.90
4	AIACQKHTPPAPKEDP	425	0.90
4	KGSPCTNVAVNPGDCP	171	0.90
5	KPPIGEHWGKGSPCTN	162	0.89
6	YFPIKKPNNNKILVPK	49	0.88
7	PYFPIKKPNNNKILVP	48	0.87
7	EHWGKGSPCTNVAVNP	167	0.87
7	RECISMDYKQTQLCLI	144	0.87
8	KVSGLQYRVFRIHLPD	64	0.86
8	HWGKGSPCTNVAVNPG	168	0.86
9	LLAVGHPYFPIKKPNN	42	0.85
9	GGTLEDTYRFVTSQAI	411	0.85
9	CAAISTSETTYKNTNF	345	0.85
9	FVTVVDTTRSTNMSLC	330	0.85
9	VGISGHPLLNKLDDTE	115	0.85
9	GQPLGVGISGHPLLNK	110	0.85
10	VFRIHLPDPNKFGFPD	72	0.84
10	TSICKYPDYIKMVSEP	226	0.84
11	NGICWGNQLFVTVVDT	321	0.83
11	GFGAMDFTTLQANKSE	204	0.83
11	GEHWGKGSPCTNVAVN	166	0.83
11	GVDNRECISMDYKQTQ	140	0.83
12	RVFRIHLPDPNKFGFP	71	0.82
12	LGKRKATPTTSSTSTT	482	0.82
12	KHTPPAPKEDPLKKYT	430	0.82
12	RHGEEYDLQFIFQLCK	365	0.82
12	PPIGEHWGKGSPCTNV	163	0.82
12	GVGISGHPLLNKLDDT	114	0.82
13	CKITLTADVMTYIHSM	379	0.81
13	TSETTYKNTNFKEYLR	350	0.81
13	GICWGNQLFVTVVDTT	322	0.81
13	ARTNIYYHAGTSRLLA	29	0.81
13	KGSGSTANLASSNYFP	278	0.81
13	LELINTVIQDGDMVDT	188	0.81
13	LCLIGCKPPIGEHWGK	156	0.81
13	DTENASAYAANAGVDN	128	0.81
14	TTSSTSTTAKRKKRKL	490	0.8
14	PPGGTLEDTYRFVTSQ	409	0.8
14	TVVDTTRSTNMSLCAA	332	0.8
14	GAMDFTTLQANKSEVP	206	0.8
14	IGEHWGKGSPCTNVAV	165	0.8
**E6**			
1	YSLYGTTLEQQYNKPL	88	0.87
2	RDLCIVYRDGNPYAVC	55	0.86
2	FHNIRGRWTGRCMSCC	132	0.86
3	TAMFQDPQERPRKLPQ	6	0.85
4	YRDGNPYAVCDKCLKF	61	0.84
4	LCIVYRDGNPYAVCDK	57	0.84
5	LKFYSKISEYRHYCYS	74	0.83
5	RWTGRCMSCCRSSRTR	138	0.83
6	YAVCDKCLKFYSKISE	67	0.82
6	DLCIVYRDGNPYAVCD	56	0.82
7	FQDPQERPRKLPQLCT	9	0.81
8	QDPQERPRKLPQLCTE	10	0.8
**E7**			
1	DGPAGQAEPDRAHYNI	39	0.85
2	AGQAEPDRAHYNIVTF	42	0.84
3	DRAHYNIVTFCCKCDS	48	0.82
3	HGDTPTLHEYMLDLQP	2	0.82
4	EEDEIDGPAGQAEPDR	34	0.81
4	EQLNDSSEEEDEIDGP	26	0.81

**Table 6:** B Cell epitopes of L1, E5, E6, & E7 proteins of HPV 18.

Rank	Sequence	Start position	Score
**L1**			
1	KPTIGPRKRSAPSATT	539	0.93
2	CQSICKYPDYLQMSAD	286	0.92
3	DNTVYLPPPSVARVVN	69	0.91
4	GLRRKPTIGPRKRSAP	535	0.9
4	KFLVQAGLRRKPTIGP	529	0.9
4	GTACKSRPLSQGDCPP	233	0.9
4	VFRVQLPDPNKFGLPD	133	0.9
4	KVSAYQYRVFRVQLPD	125	0.9
5	AITCQKDAAPAENKDP	487	0.87
5	RHVEEYDLQFIFQLCT	427	0.87
5	CASTQSPVPGQYDATK	406	0.87
5	LGCAPAIGEHWAKGTA	220	0.87
6	TVVDTTPSTNLTICAS	393	0.86
6	NGVCWHNQLFVTVVDT	382	0.86
6	GSCVYSPSPSGSIVTS	348	0.86
6	QSICKYPDYLQMSADP	287	0.86
6	GVGLSGHPFYNKLDDT	175	0.86
6	VSAYQYRVFRVQLPDP	126	0.86
7	TPTSIFYHAGSSRLLT	90	0.85
7	KGTACKSRPLSQGDCP	232	0.85
7	HWAKGTACKSRPLSQG	229	0.85
8	QMALWRPSDNTVYLPP	61	0.84
8	SIVTSDSQLFNKPYWL	359	0.84
8	KGTGMPASPGSCVYSP	339	0.84
8	RHFWNRAGTMGDTVPQ	319	0.84
8	IGEHWAKGTACKSRPL	226	0.84
8	LGVGLSGHPFYNKLDD	174	0.84
9	MSYIHSMNSSILEDWN	450	0.83
9	ICASTQSPVPGQYDAT	405	0.83
9	VGLSGHPFYNKLDDTE	176	0.83
10	AAPAENKDPYDKLKFW	494	0.82
10	PAIGEHWAKGTACKSR	224	0.82
10	GVEIGRGQPLGVGLSG	165	0.82
10	DTSIYNPETQRLVWAC	148	0.82
10	TVGNPYFRVPAGGGNK	105	0.82
11	TVYLPPPSVARVVNTD	71	0.81
11	TIGPRKRSAPSATTSS	541	0.81
11	AGLRRKPTIGPRKRSA	534	0.81
11	NSSILEDWNFGVPPPP	457	0.81
11	SVDYKQTQLCILGCAP	209	0.81
11	SHAATSNVSEDVRDNV	193	0.81
11	RVQLPDPNKFGLPDTS	135	0.81
12	PPSVARVVNTDDYVTP	76	0.8
12	PAENKDPYDKLKFWNV	496	0.8
12	SEDVRDNVSVDYKQTQ	201	0.8
**E5**			
1	CFCVCMYVCCHVPLLP	9	0.86
2	PATAFTVYVFCFLLPM	46	0.83
**E6**			
1	HNIAGHYRGQCHSCCN	128	0.89
2	LQDIEITCVYCKTVLE	25	0.86
3	TRRPYKLPDLCTELNT	8	0.82
3	HKCIDFYSRIRELRHY	66	0.82
4	AGHYRGQCHSCCNRAR	131	0.81
4	NIAGHYRGQCHSCCNR	129	0.81
5	RRPYKLPDLCTELNTS	9	0.8
**E7**			
1	KATLQDIVLHLEPQNE	5	0.82

**Table 7:** Predicted B cell epitopes in vaccine construct.

S. No	Rank	Sequence	Start position	Score
1	1	LQFIFAAYKFGFPDTS	105	0.90
2	2	PGSIFYHAGSSRLLTV	357	0.89
3	3	PGTAFTVYVFCFLLPM	397	0.88
4	3	QERPRKLAAYRAHYNI	151	0.88
5	4	PGPGVMTYIHSMNSTI	315	0.86
6	4	EEQIGKCSTRGRKCCR	27	0.86
7	5	HIHAIAAYKLPDLCTE	261	0.85
8	6	LVAAYDPQERPRKLAA	144	0.83
9	6	YCRVRGGRCAVLSCLP	10	0.83
10	7	VGGPGPGPLGRKFLVQ	372	0.82
11	7	IVTFAAYEPDRAHYNI	166	0.82
12	8	AGLRRKGPGPGTAFTV	388	0.81
13	8	AGSSRLLTVGGPGPGP	364	0.81
14	8	AVLSCLPKEEQIGKCS	19	0.81
15	9	LFLIHTHARFLIGPGP	342	0.80
16	9	YLTVGNPYFRAAYLYH	220	0.80

### Multi-epitope chimeric vaccine construction and its characterization

**Figure 2:**
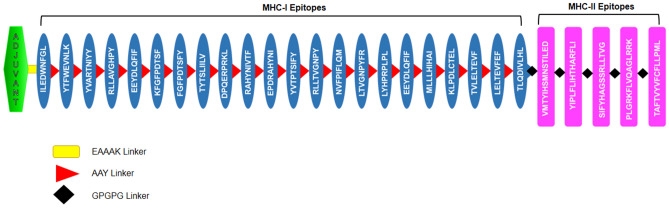
Schematic representation of the prepared multi-epitope chimeric vaccine construct having adjuvant, MHC-I & MCH-II epitopes linked with EAAAK, AAY & GPGPG linkers respectively.

**Figure 3:**
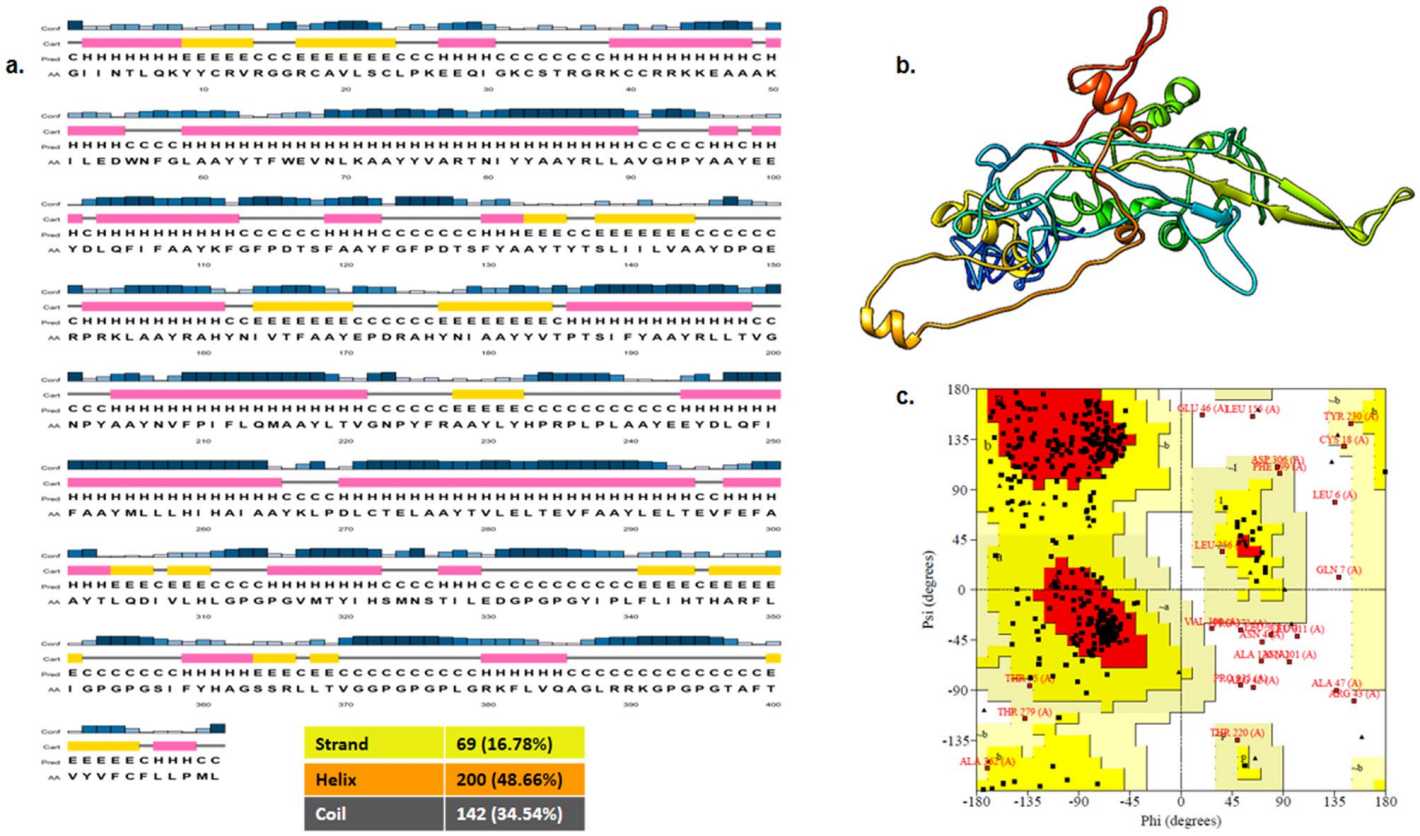
Predicted 2D and 3D structure of the construct, (**a**). Predicted 2D structure of the multi-epitope chimeric vaccine construct, (**b**). Predicted 3D structure of the multi-epitope chimeric vaccine construct by the online I-TASSER server (https://zhanglab.ccmb.med.umich.edu/I-TASSER). The 3D structure of the protein was visualized in UCSF Chimera version1.13rc https://www.rbvi.ucsf.edu/chimera. (**c**). Ramachandran Plot analysis of the multi-epitope chimeric vaccine construct.

The potent epitopes of L1, E5, E6 & E7 proteins of HPV 16 & 18 were selected and joined with specific linkers for the adjuvant, MHC class I and MHC class II epitopes. In the vaccine construct, adjuvant β-defensin, 45 amino acid long, was at the N terminal and linked to the MHC-I alleles with EAAAK linker. The MHC-I epitopes of L1, E5, E6 & E7 proteins of HPV 16 & 18 were linked together with the AAY linker, on the other hand MHC-II epitopes were linked with GPGPG linkers and the final construct was prepared. Final vaccine construct has the 411 amino acid along with the addition of the adjuvant and linkers. The schematic representation of the prepared construct along with the secondary and tertiary structure shown in Figs. 2 and 3, respectively.

### Physiochemical properties of multi-epitope chimeric vaccine construct favored for vaccine production

The physiochemical properties of the multi-epitope chimeric **v**accine construct were analyzed using the ProtParam server. The 411 amino acid of this construct has a molecular weight of around 46,246.53 g/mol and theoretical pI was 8.67, depicting it as basic nature. The construct is thermostable as its aliphatic index is 91.07 and average half-life in 30 h *in-vivo* (in mammalian reticulocytes), whereas > 20 & > 10 h in yeast & *Escherichia coli (E. coli)*, respectively.

### Allergenicity and antigenicity

The allergenic property of the multi-epitope chimeric vaccine construct was predicted by AllerTOP v2.0 and found to be non-allergenic behavior. The predicted antigenicity score of this construct was 0.5883 by using Vaxijen v2.0 at a threshold value of > 0.4%, which shows the antigenic nature of the construct.

### The multi-epitope chimeric vaccine construct favored secondary and tertiary structure

The 411 amino acid long multi-epitope chimeric vaccines construct was analyzed for secondary structure, out of 411 amino acids, 200 (48.66%) formed alpha helix, 69 (16.78%) β-strands and coil were formed by 142 (34.54%) (Fig. 3a). I-TASSER uses 10 threading templates which showed good Z-score values (ranging from 0.62 to 3.54) and predicted 5 models. The model was selected on the basis of a high C score (higher value indicates higher confidence) which typically ranges from − 5 to 2 (Fig. 3b). The selected model was analyzed for the Ramachandran plot using the SAVES server and found 93.8% of residues in the most favored regions and additional allowed regions (Fig. 3c).

### Toll-like receptor (TLR)-3 and TLR4 established the interaction with multi-epitope chimeric vaccine construct

**Figure 4:**
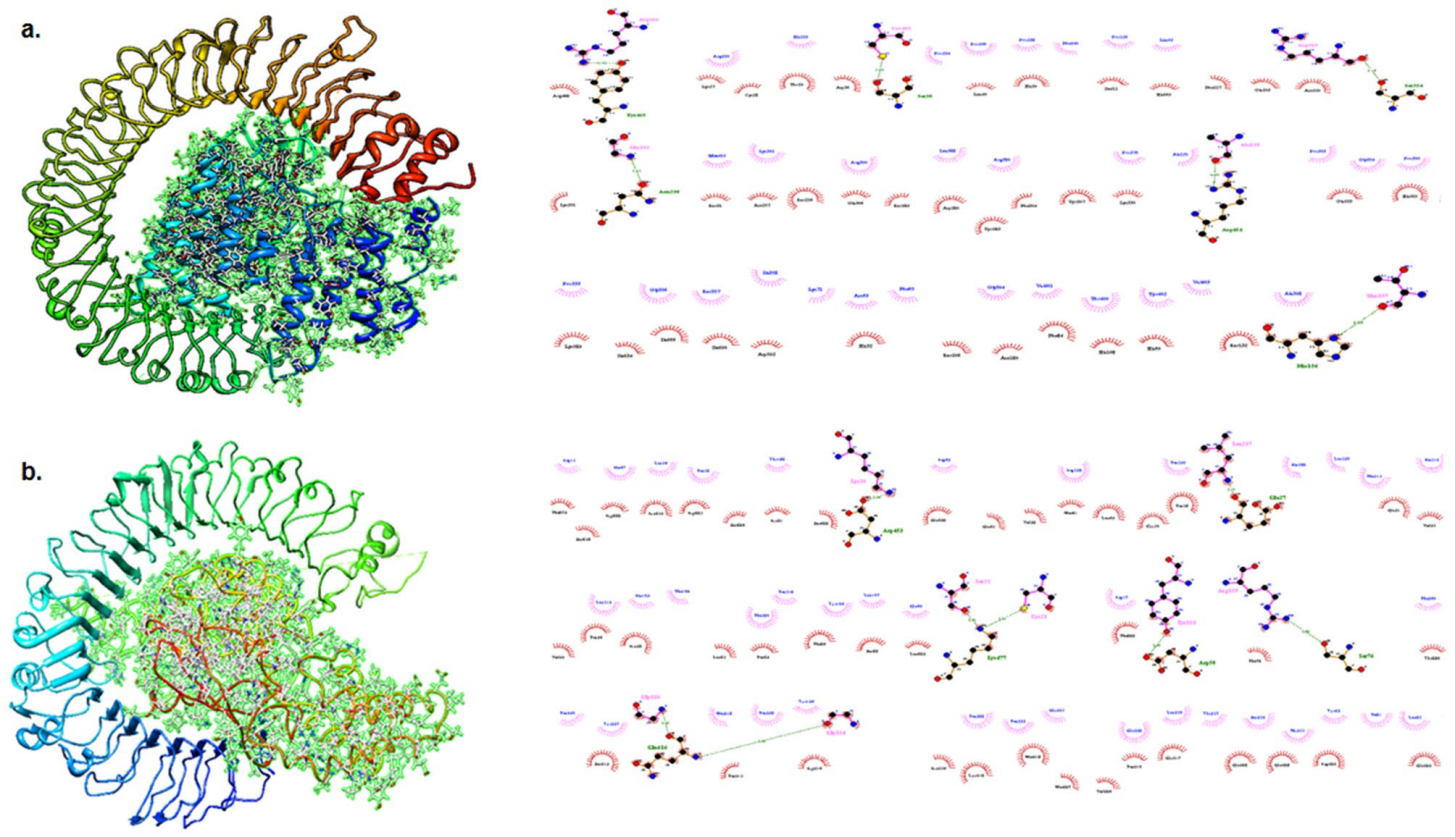
Molecular docking between multi-epitope chimeric vaccine construct and TLR 3 & TLR 4 receptors using the PatchDock (https://bioinfo3d.cs.tau.ac.il/PatchDock/php.php) server, (**a**). Depiction of the docked conformation and interaction of the TLR 3 & multi-epitope chimeric vaccine construct in Ligplot analysis indicating hydrogen bonding and hydrophobic interactions between the construct and TLR3, (**b**). Depiction of the docked conformation and interaction of the TLR 4 & multi-epitope chimeric vaccine construct in Ligplot analysis indicating hydrogen bonding and hydrophobic interactions between the construct and TLR4.

The multi-epitope chimeric vaccine construct with the selected epitopes was docked with TLR3 (2A0Z) and TLR4 (3FXI) and showed good interaction. The docked structure along with molecular interactions between docking complex of vaccine construct and TLR3 & TLR4 were visualized in the LigPlot (Fig. 4a,b).

### MD simulation studies show a stable interaction between ligand and its receptor

**Figure 5:**
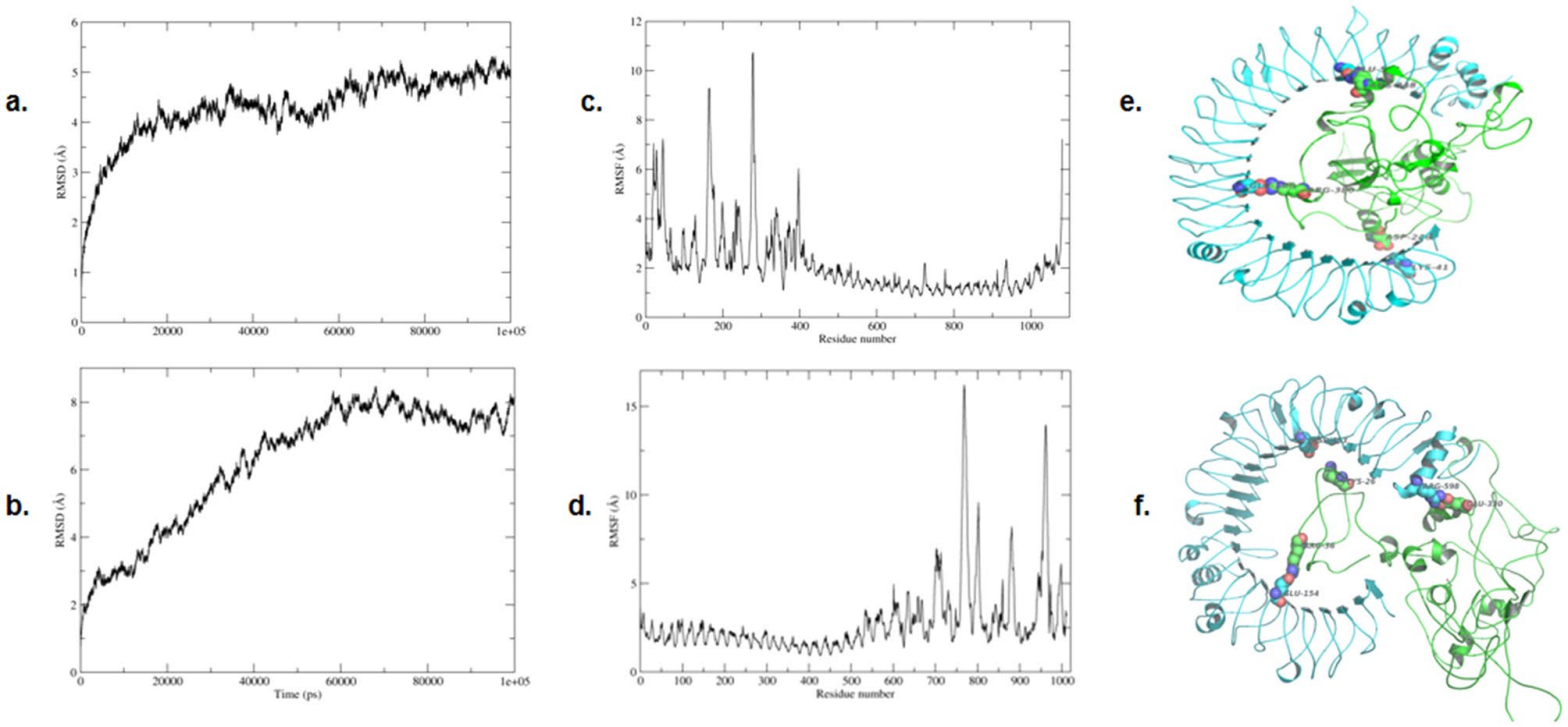
MD Simulation Results. (**a**). RMSD curve for TLR3-chimeric vaccine construct complex, (**b**). RMSD curve for TLR4-chimeric vaccine construct complex, (**c**). RMSF curve for TLR3-chimeric vaccine construct complex, (**d**). RMSF curve for TLR4-chimeric vaccine construct complex. The beta sheets of the TLR3 and TLR4 show limited flexibility. The flexibility of the interface binding residues is also less indicating stable interactions, (**e**). The complex of TLR3-chimeric vaccine construct complex generated by the VMD software version 1.93^57^, (**f**). The complex of TLR4-chimeric vaccine construct complex generated by the VMD software version 1.93^57^, Three most stable salt bridges are shown for both of the complexes. The salt bridges anchor the two proteins by stable salt-bridge interactions. The complex of TLR3-chimeric vaccine construct showed stronger salt-bridge interactions as measured by occupancy indicating it may be comparatively more stable than TLR4-chimeric vaccine construct complex.

The molecular dynamics analysis was performed to access the binding interactions and flexibility of the binding site. MD simulations were done for 100 ns on each complex (total 200 ns) and the stability of the simulation was evaluated using root mean square deviation (RMSD). The RMSD values reveal the structural changes that occurred during the MD. The RMSD plots for all proteins indicated that each system got stabilized quickly and then remained stable throughout the simulation time as evidenced by the movement of the RMSD curve within the 2 Å (Fig. 5a,b). These plots suggested that each system was quite stable for further study.

The root-mean-square fluctuation (RMSF) study was done to get an insight into the flexibility of individual amino acids during the simulation. The amino acid residues taking part in important interactions are generally constrained and show less flexibility as compared to other amino acid residues in the complex. It can be seen from RMSF plots (Fig. 5c,d) that the beta-sheets of TLR3 and TLR4 are showing less fluctuations (wavy pattern) due to strong hydrogen bonding between them. The other part of HPV is showing greater fluctuations. A comparison of the two plots indicates that the complex with TLR3 is showing comparatively lesser fluctuations indicating better binding among the interaction partners and a stronger complex.

The chimeric vaccine construct-TLR3 salt-bridge analysis revealed residues at the binding interface show strong interactions especially ARG348_chimeric vaccine construct-GLU533_TLR3, ASP246_chimeric vaccine construct-LYS41_TLR3, ARG380_chimeric vaccine construct-GLU306_TLR3 were found to be quite stable with percent occupancy of 72, 63 and 55 respectively. It can be seen in Fig. 5e that these salt bridges anchor the two proteins at three distinct points. In case of HPV-TLR4 complex, the binding interactions were less strong as compared with the chimeric vaccine construct-TLR3 complex. The ARG598_TLR4-GLU330_chimeric vaccine construct, ASP453_TLR4-LYS26_chimeric vaccine construct, GLU154_TLR4-ARG36_chimeric vaccine construct interactions showed however some stabilizing interactions with > 40% occupancy. The salt-bridges are shown in Fig. 5f. The results clearly showed the complex HPV-TLR3 is much more stable than chimeric vaccine construct-TLR4.

### In silico cloning of the construct in pcDNA3.1/V5/His-Topo vector

**Figure 6:**
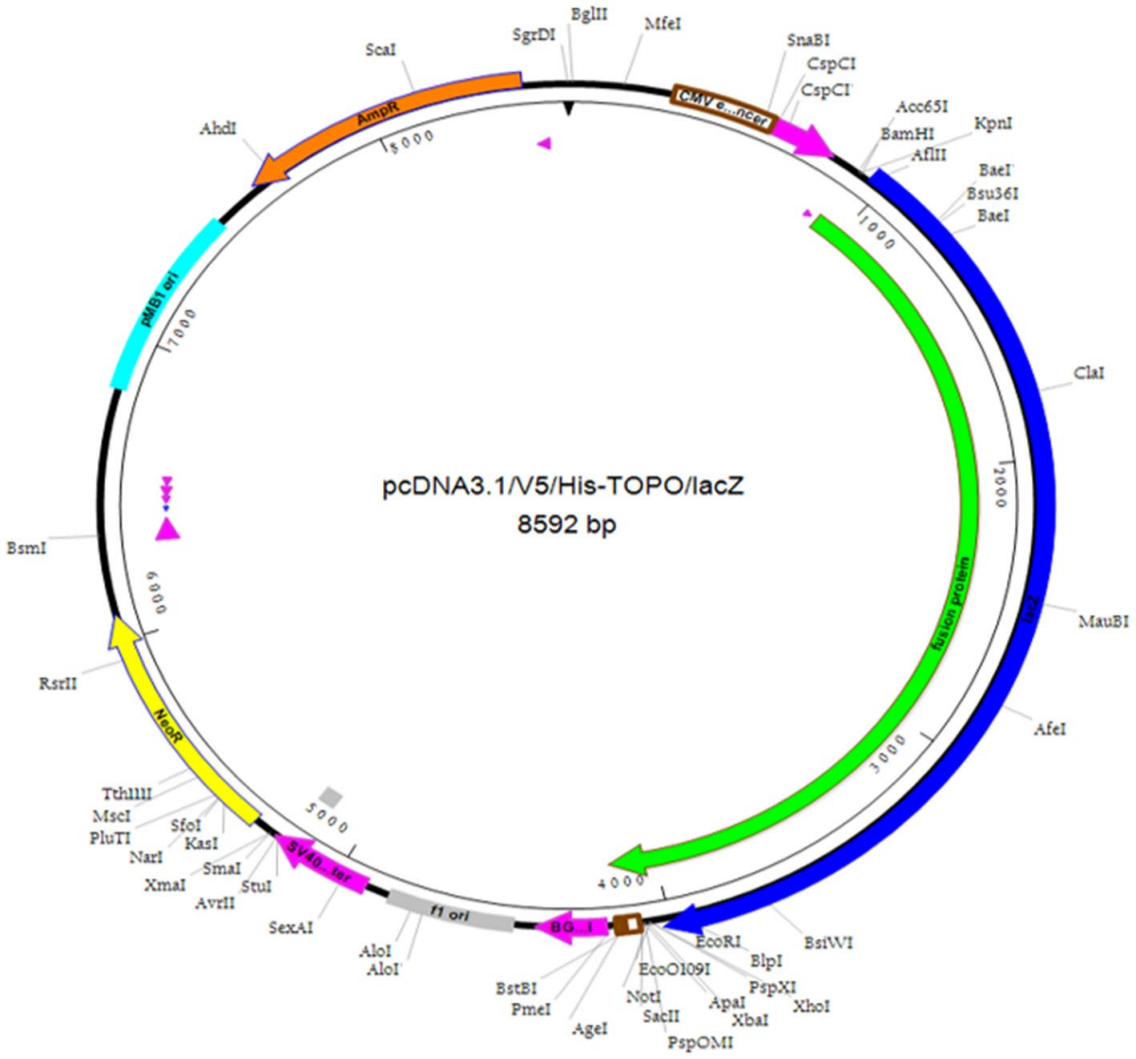
In silico cloning of multi-epitope chimeric vaccine (shown in green) in pcDNA3.1/V5/His-TOPO/LacZ vector.

The vaccine candidate was processed for the codon optimization for the maximal level of protein expression in *E. coli* using Jcat server and the optimized vaccine candidate had 1233 nucleotide and the GC content of 67.8%, which showed the high expression of the vaccine candidate. The plasmid vector was prepared using the SnapGene software into pcDNA3.1/V5/His-Topo (Fig. 6).

### Immune simulation analysis shows an effective cytokine and antibody response

**Figure 7:**
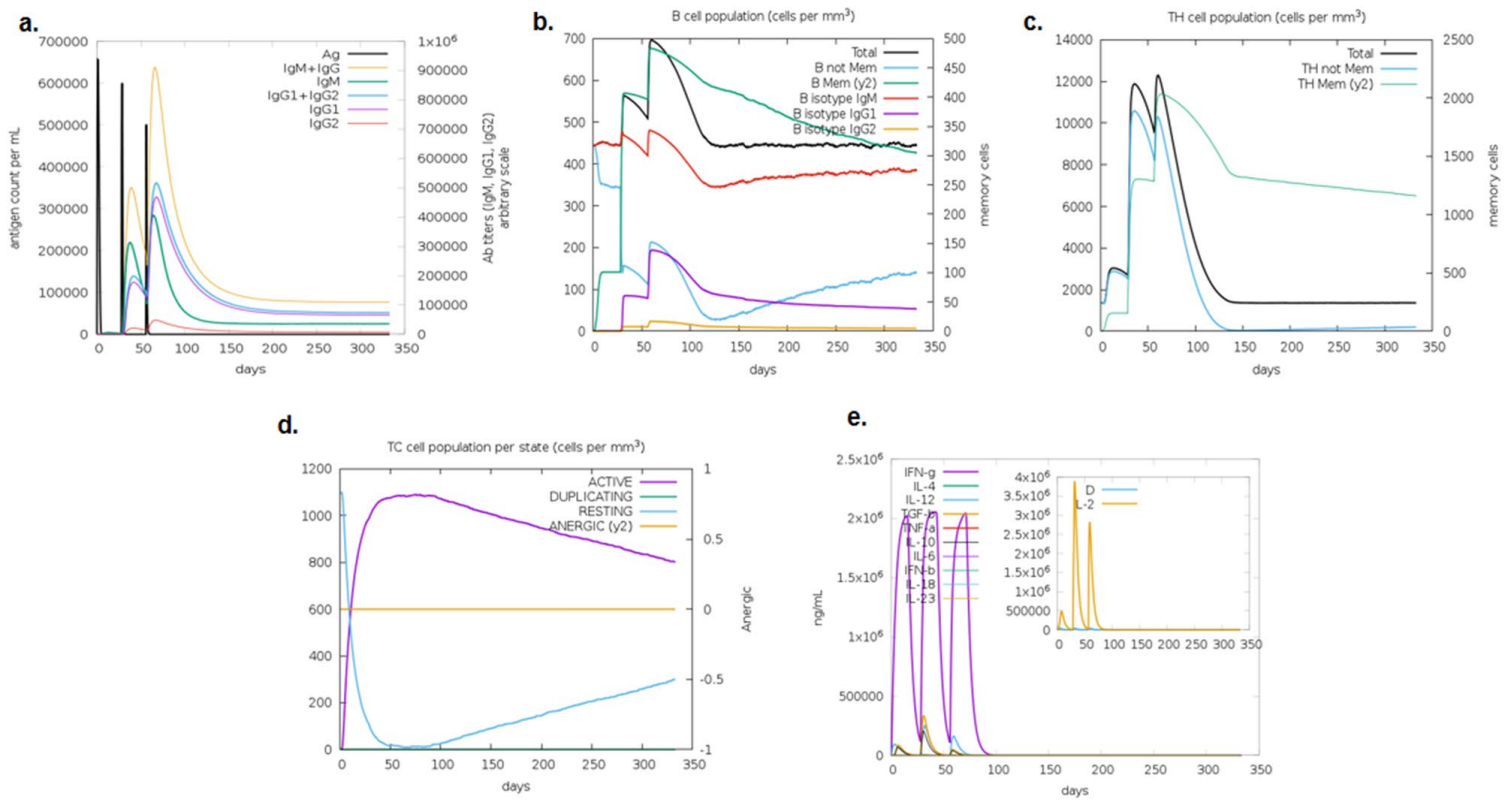
Immune stimulation using in silico C-ImmSim server after antigen 3 dose injection of vaccine construct^61^, (**a**). Production of the immunoglobulin after antigen injections (black vertical lines); detailed subclasses are showed as coloured peaks, (**b**)**.** Evolution of B-cell populations, (**c**). Th cell population evolution, (**d**). T-cytotoxic cell populations, (**e**). Production of cytokines & interleukins.

The result of the C-ImmSim studies showed the effective immune responses of the vaccine candidate which showed the high level of the IgM and also showed the increased level of the immunoglobulin (IgG1 + IgG2, IgM and IgG + IgM) expression and T helper cell and cytotoxic T cells. The production of the cytokines & interleukins was identified along with increased B- & T-cells (Fig. 7).

## Discussion

Cervical cancer caused by HPV has reached to its alarming stage and become a public threat globally^14^. The current management of this disease is not satisfactory and mainly depends on three prophylactic vaccines^15^. So far, no therapeutic vaccine is available against HPV. Hence, a vaccine having both prophylactic and therapeutic properties will help not only to prevent the HPV infection but should be used to treat the established infection.

Nowadays, immunoinformatics, a branch of bioinformatics, offers new tools for the identification and design of epitopes against specific antigens, which could be used as an ideal target for the development of vaccine against specific viral or pathogenic infections^16,17^. Immunoinformatics, helps in the identification of effective epitopes which can activate both cell-mediated or humoral immunity against specific viral or pathogenic infections. In 2003, Adu-Bobie et al., developed the first vaccine against *Neisseria meningitides* by using immunoinformatics approach^18^. Many research articles showed the importance of the highly immunogenic epitopes for CD8 + and CD4 + CTLs is required in a vaccine for persuading a strong immune response^19,20^. The peptide (generally 8–11 residues) presented by the MHC-I molecules intracellularly recognized by CD8 + CTLs, whereas the MHC-II recognized extracellular-originated peptides (generally 10–30 residues) by CD4 + Helper T lymphocytes (HTLs). The ideal peptide length for MHC-I and MHC-II is 9 residue & 12–16 residue, respectively. The strong immune response generated by a vaccine depends on the interaction of peptide-MHC complex (pMHC) and T cell receptor^19,21^. Also, This reverse vaccinology approach has various advantages due to its less time-consuming property, cost-effectiveness, more accuracy and safety. Therefore, most of the research has been done taking this approach in different organisms such as viruses, bacteria, and parasites, for the identification and development of multi-epitope vaccines^22–29^.

In the present study, the epitopes for the MHC-I & MHC-II were predicted for the L1, E5, E6 & E7 proteins of two highly oncogenic strains of HPV i.e. HPV 16 & HPV 18. These selected proteins of HPV 16 & HPV 18 play an important role in the viral structure, cell transformation and immune evasion to induce malignancy in the cells^30–32^. The chimeric vaccine construct prepared in the study has also shown 16 potential B cell epitopes when submitted to ABCpred server. The most potent epitopes were LQFIFAAYKFGFPDTS, PGSIFYHAGSSRLLTV, & PGTAFTVYVFCFLLPM having score more than 0.90, 0.89 & 0.88, respectively. Further we have made multi-epitope chimeric construct with the help of adjuvants and linkers with appropriate position.

The final vaccine construct has the molecular weight of 46.24 kDa with chemical formula C_2177_H_3223_N_529_O_564_S_13_ and has a theoretical isoelectronic point (pI) was 8.67, which suggested the basic nature of the prepared vaccine construct. The aliphatic index and grand average of hydropathicity (GRAVY) of the construct were 91.07 and 0.186, respectively, which suggested that protein is thermostable & hydrophilic in nature. Sympathetic physiochemical properties of the vaccine construct suggested it as a functional vaccine candidate. Knowing the structural properties like secondary & tertiary structure of the vaccine candidate is important for a vaccine candidate. On analyzing, the vaccine candidate has 48.66% alpha-helix, 16.78% β-strands and 34.54% coil. The protein or peptide having alpha helix & coiled coils are important as structural antigens as they can be recognized by antibodies. The 3D structure of the vaccine construct showed the 93.8% of residues in the most favoured regions and additional allowed regions, which indicate the acceptable quality of the predicted vaccine candidate. The multi-epitope chimeric construct was antigenic and non-allergic suggesting its efficacy for the induction of robust immune responses without any allergic responses. The docking results of vaccine construct with TLR3 & TLR4 showed a high binding affinity towards TLR3 & TLR4.

Further, the immune simulation showed an increased T & B cell production. In addition, the B-cells were present for several months along with the stimulated T helper cells. The expression of cytokines showed the increased level of IFN-γ from first injection and gradually reached to the peak level after repeated injection i.e., exposure of the antigen. The increased level of the T helper cells showed the efficient production of the immunoglobulin (Ig) to support humoral response. The expression of the vaccine construct is important in the suitable host. The vaccine construct was optimized and has 67.79% of GC content and the codon CAI-value of the improved sequence was 0.95, which indicates favourable expression like *E.coli* (Rosano, G. L. & Ceccarelli, E. A. et al., 2014). The optimized sequence was cloned in the pcDNA3.1/V5/His-Topo for efficient expression.

Earlier, several attempts have been made for the development and identification of multi-epitope vaccines against HPV such as the use of virus-like particles (VLPs) based on L1 protein^33^ and L2-based vaccination^34,35^. In another study by Negahdaripour et al., predicted epitopes from the HPV 16 L2 protein^36,37^ while other studies used L1 protein of HPV 16 for epitope prediction^38^. Overall, most of the studies were performed using E6 and E7 protein as key molecules for epitope prediction due to various reasons.

The search for a better vaccine candidate is still under consideration. Earlier, most of the studies were done by targeting E6 and E7 proteins of HR-HPV 16 and 18. However, none of them reached clinical trials. The majority of the previous studies have focused on the E6, E7 oncoproteins and L1 capsid protein of HPV. However, apart from the oncogenic E6, E7 protein and highly immunogenic L1 protein, E5 is crucial in HPV pathogenesis. E5, along with E6 and E7 is responsible for the transformation of normal cells, downregulation of MHC I expression, protein trafficking through ER and preventing the acidification of endosomes^39–43^ Recently, Namvar et al. predicted epitopes from the E5 and E7 proteins of HPV 16/18/31/45^44^ implying the future potential of targeting HPV E5 protein.

Here, for the first time we have analyzed and taken the major oncoproteins (E6 and E7), highly immunogenic L1 capsid protein and also, E5 protein from both the oncogenic strains of HPV (HPV16 &18) and prepared a multi-epitope chimeric vaccine through in silico approach. Our multi-epitope construct has peptides like CYSLYGTTL, ISEYRHYCY, KLPDLCTEL, LLMGTLGIV and TLQDIVLHL that have been experimentally validated^45–49^. In addition, the first HPV 16 E7 predicted epitope in our study RAHYNIVTF, was also recognized by Feltkamp et al. 1993 who were the pioneers of HPV epitope studies^50^.

Taken together, our results showed the chimeric construct has the ability to induce both cell-mediated and humoral response and consisting all immunogenic, physicochemical and structural properties which are required for ideal vaccine design. The immune stimulation analysis suggested the efficiency of our construct to initiate an effective cytokine response and B cell response. Finally, our multi-epitope chimeric construct can be used in both prophylactic and therapeutic purposes in *in-vitro*/*in-vivo* studies against HPV infection. In addition, further validation will be required for the use of our construct in combination with other cancer immunotherapy approaches to target HPV-induced malignancies.

## Material and method

### Retrieving of protein sequences

The amino acid sequences of L1, E5, E6 & E7 proteins of HPV 16 (NP_041332; NP_041330; NP_041325 & NP_041326) and HPV 18 (NP_040317; NP_040315; NP_040310; & NP_040311) were retrieved from the National Center for Biotechnology Information (https://www.ncbi.nlm.nih.gov/).

### T-cell epitopes prediction

The epitopes for MHC-I & MHC-II alleles from retrieved protein sequences of L1, E5, E6 & E7 proteins of HPV 16 & 18 were predicted by using Immune Epitope Database (IEDB): analysis resource (http://www.iedb.org/) supported by National Institute of Allergy and Infectious Diseases, a component of the National Institutes of Health in the Department of Health and Human Services. The default parameters were used for the prediction of the epitopes using the IEDB server. The percentile score (lower the percentile value higher the binding affinity of the predicted epitope) was chosen less than 0.5 for MHC-I alleles and 0.5 for MHC-II alleles.

### Prediction of MHC Class I immunogenicity

The identified MHC class I epitopes were further examined and confirmed for the immunogenicity by using another tool i.e. IEDB MHC Class I immunogenicity (http://tools.iedb.org/immunogenicity/). This tool only analyzed and validated on 9 mer peptides.

### Prediction of antigenicity & IFN-γ inducing epitope

Vaxijen, an online tool was used to check the antigenicity in predicted epitopes^51^. Further, to confirm whether selected epitopes have the ability to induce IFN-γ, we have used IFN epitope server (https://crdd.osdd.net/raghava/ifnepitope/predict.php) in this study^52^. In our analysis, we have performed Motif and SVM hybrid algorithms and IFN-γ versus non IFN-γ model for prediction^52^.

### Preparation and Physiochemical properties of the vaccine construct

A chimeric vaccine construct of HPV 16 and HPV 18 having MHC class I & II predicted immunogenic and conserved epitopes were analyzed together with linkers to get the final vaccine construct using β-defensin adjuvant. It has been well established the role and properties of β-defensin as an adjuvant against viral infections^24,53^, which was associated with EAAAK linker at N terminal. The AAY linker was associated with MHC- I epitopes while MHC-II epitopes were linked with GPGPG linkers. The Protparam server was used to compute and validate the physiochemical properties of the final vaccine construct.

### Prediction of B-cell epitopes

In the reverse vaccinology approach, a successful vaccine must also induce a strong B-cell mediated humoral immune response. Hence, it is of utmost to show that chimeric vaccine construct is able to induce protective humoral immunity. Here, for B cell epitope prediction the artificial neural network-based ABCpred server (http://www.imtech.res.in/raghava/abcpred/ABCsubmission.html) was employed. All the parameters were setup in default conditions but selected the epitope having score more than 0.8^54^.

### Prediction of Allergenicity

Proteins and peptides are well known to induce allergenic reaction. We have used an online tool AllerTOP v2.0 (https://www.ddg-pharmfac.net/AllerTOP/) to check the allergenicity of the chimeric vaccine construct.

### Prediction of secondary and tertiary structure

The Psipred, which is an online server was used to predict the secondary structure of prepared chimeric vaccine construct which mainly uses primary amino acid sequences in specific manner (http://bioinf.cs.ucl.ac.uk/psipred/). For the prediction of the tertiary structure of the chimeric vaccine construct, the online freely available server I-TASSER server (https://zhanglab.ccmb.med.umich.edu/I-TASSER/) was used which utilizes sequence-to-structure-to-function paradigm. The last five community-wide CASP experiments ranked I-TASSER server as the top server for the prediction of protein structure^24,55^.

### Validation of the tertiary structure

PDBsum, a pictorial database was used to validate the tertiary structure which is based on to generate Ramachandran plot (http://www.ebi.ac.uk/thornton-srv/databases/pdbsum/Generate.html) that gives an at-a-glance impression of each 3D structure contents submitted in the PDB. Finally, the validation of the 3D structure is vital for the protein model, as it can detect potential errors in modeled 3D structure.

### Molecular docking of vaccine construct with TLR3 and TLR4

One of the most crucial steps in reverse vaccinology is to check and predict the chimeric vaccine construct efficacy by molecular docking. Here we have docked TLR 3 (2A0Z) & TLR4 (3FXI) with prepared chimeric vaccine construct by using PatchDock (https://bioinfo3d.cs.tau.ac.il/PatchDock/php.php) server. This server is very efficient and fast and works on specific algorithms. Another server FireDock was used to refine and for the re-scoring of the results obtained from docking (http://bioinfo3d.cs.tau.ac.il/FireDock/php.php). This server generates better results with the lowest global energy. Finally, the best-docked model was used and selected and a visualizing tool UCSF Chimera was used for biocomputing, visualization and informatics for analysis^56^.

### MD simulation

In the current study, the preliminary topology and coordinates for the complexes were generated in VMD version 1.93^57^. The complexes were prepared and solvated in a rectangular water box (TIP3P) with a buffering distance of 10 Å. Ions were added to ensure the electro-neutrality of the solvated system. SETLE algorithm was used for the water molecules model system. The associated system topology and coordinates were generated by applying charmm34 force field parameters for MD simulation. The MD simulations were executed using NAMD version1.9^58^. Prior to the simulation, the system was properly minimized with a stepwise minimization protocol. Firstly, the water molecules and ions were minimized, followed by hydrogen atoms and the side chains of the complex. The side chains were minimized for 100,000 steps while the backbone atoms and the bond lengths of hydrogen atoms were kept fixed. Thereafter, all the atoms were allowed to relax freely and the whole system was energy-minimized for 100,000 steps with nominal restraints on C-alpha atoms and DNA backbone atoms (10 kcal/mol) to prevent any abrupt change in structure. Subsequently, an equilibration protocol was followed where the system was heated gradually from 0–310 K in steps of 30 K with a canonical ensemble (NVT). At each step, a 20 picosecond (ps) simulation was run to allow the system to adjust to the temperature. Once the system attained 310 K, an isobaric and isothermic ensemble (NPT) was applied for a period of 100 ps with a constant pressure of 1.0 bar using Langevin dynamics0. Finally, the applied restraints on C-alpha atoms and DNA were removed and the system was equilibrated for 1 ns at 310 K using Langevin piston coupling algorithm. During the whole simulation, the Particle Mesh Ewald sum algorithm (PME) algorithm was used to calculate the long-range electrostatic interactions, the hydrogens were constrained using SHAKE algorithm. After equilibration, a production run was done on each complex for 100 ns. The analyses of the MD trajectories were performed to get an insight on the structure and dynamic behavior of all complexes. The trajectories were analysed for root mean square deviation (RMSD), root mean square fluctuation (RMSF), hydrogen bonds, and salt bridges in VMD.

### In silico cloning and optimization of designed vaccine candidate

Java Codon Adaptation Tool (JCat) server (http://www.jcat.de/) was used for codon optimization and reverse translation to check translation and efficiency of cloning of the multi-epitope vaccine construct. The protein expression level was assessed by using codon adaptation index (CAI) and the percentage GC content generated in JCat output^59^. The optimized sequence of the vaccine was then cloned into pcDNA3.1/V5/His-TOPO/LacZ vector using DNASTAR (https://www.dnastar.com).

### Immune simulation

In order to characterize the effective immune response generated by the prepared vaccine construct, an online simulation server, C-ImmSim (http://150.146.2.1/C-IMMSIM/index .php), was used^61^. This server uses a position-specific scoring matrix (PSSM) to predict immune epitope and predict the immune interaction by using machine learning. It predicted both cellular and humoral immune in mammalian immune system^60,61^. The server was used on the default parameter with simulation volume 50 and the simulation steps 1000 with three injections of the predicted vaccine construct at an interval of 4 weeks.

## References

[1] Brüggmann D (2018). Human papilloma virus: global research architecture assessed by density-equalizing mapping. Oncotarget.

[2] Forman D (2012). Global burden of human papillomavirus and related diseases. Vaccine.

[3] Doorbar J, Egawa N, Griffin H, Kranjec C, Murakami I (2015). Human papillomavirus molecular biology and disease association. Rev. Med. Virol..

[4] Zur Hausen H (2009). Papillomaviruses in the causation of human cancers—a brief historical account. Virology.

[5] Parkin DM, Bray F (2006). Chapter 2: The burden of HPV-related cancers. Vaccine.

[6] de Martel C, Plummer M, Vignat J, Franceschi S (2017). Worldwide burden of cancer attributable to HPV by site, country and HPV type. Int. J. Cancer.

[7] Li J (2017). A novel therapeutic vaccine composed of a rearranged human papillomavirus type 16 E6/E7 fusion protein and Fms-like tyrosine kinase-3 ligand induces CD8+ T cell responses and antitumor effect. Vaccine.

[8] Manuri PR (2007). Intranasal immunization with synthetic peptides corresponding to the E6 and E7 oncoproteins of human papillomavirus type 16 induces systemic and mucosal cellular immune responses and tumor protection. Vaccine.

[9] Schiller JT, Castellsagué X, Garland SM (2012). A review of clinical trials of human papillomavirus prophylactic vaccines. Vaccine.

[10] Joura EA (2015). A 9-Valent HPV vaccine against infection and intraepithelial neoplasia in women. N. Engl. J. Med..

[11] Bian H, Reidhaar-Olson JF, Hammer J (2003). The use of bioinformatics for identifying class II-restricted T-cell epitopes. Methods (San Diego, Calif).

[12] Li GF (2005). Identification of immunodominant Th1-type T cell epitopes from Schistosoma japonicum 28 kDa glutathione-S-transferase, a vaccine candidate. Acta Biochim. Biophys. Sin..

[13] Kohaar I (2009). Association between human leukocyte antigen class II alleles and human papillomavirus-mediated cervical cancer in Indian women. Hum. Immunol..

[14] Wang X, Huang X, Zhang Y (2018). Involvement of human papillomaviruses in cervical cancer. Front. Microbiol..

[15] Madrid-Marina V, Torres-Poveda K, López-Toledo G, García-Carrancá A (2009). Advantages and disadvantages of current prophylactic vaccines against HPV. Arch. Med. Res..

[16] Tong JC, Ren EC (2009). Immunoinformatics: current trends and future directions. Drug Discov. Today.

[17] Cohen, T., Moise, L., Martin, W. & De Groot, A. S. in *Infectious Disease Informatics* (ed Vitali Sintchenko) 223–244 (Springer New York, 2010).

[18] Masignani V (2003). Vaccination against Neisseria meningitidis using three variants of the lipoprotein GNA1870. J. Exp. Med..

[19] Gratz IK (2014). Cutting edge: Self-antigen controls the balance between effector and regulatory T cells in peripheral tissues. J. Immunol..

[20] Schneidewind A (2008). Structural and functional constraints limit options for cytotoxic T-lymphocyte escape in the immunodominant HLA-B27-restricted epitope in human immunodeficiency virus type 1 capsid. J. Virol..

[21] Rammensee HG, Bachmann J, Emmerich NPN, Bachor OA, Stevanović S (1999). SYFPEITHI: database for MHC ligands and peptide motifs. Immunogenetics.

[22] Enayatkhani M (2021). Reverse vaccinology approach to design a novel multi-epitope vaccine candidate against COVID-19: an in silico study. J. Biomol. Struct. Dyn..

[23] Solanki V, Tiwari M, Tiwari V (2019). Prioritization of potential vaccine targets using comparative proteomics and designing of the chimeric multi-epitope vaccine against Pseudomonas aeruginosa. Sci. Rep..

[24] Shey RA, Ghogomu SM, Esoh KK, Nebangwa ND, Shintouo CM, Nongley NF, Souopgui J (2019). In-silico design of a multi-epitope vaccine candidate against onchocerciasis and related filarial diseases. Sci. Rep..

[25] Khan A (2019). A systems Vaccinology approach reveals the mechanisms of immunogenic responses to Hantavax vaccination in humans. Sci. Rep..

[26] Khatoon N, Pandey RK, Prajapati VK (2017). Exploring Leishmania secretory proteins to design B and T cell multi-epitope subunit vaccine using immunoinformatics approach. Sci. Rep..

[27] Kar T (2020). A candidate multi-epitope vaccine against SARS-CoV-2. Sci. Rep..

[28] Khalid H, Ashfaq UA (2020). Exploring HCV genome to construct multi-epitope based subunit vaccine to battle HCV infection: Immunoinformatics based approach. J. Biomed. Inform..

[29] Akhtar N, Joshi A, Kaushik V, Kumar M, Mannan MA-U (2021). In-silico design of a multivalent epitope-based vaccine against Candida auris. Microb. Pathog..

[30] Howie HL, Katzenellenbogen RA, Galloway DA (2009). Papillomavirus E6 proteins. Virology.

[31] DiMaio D, Petti LM (2013). The E5 proteins. Virology.

[32] Roman A, Munger K (2013). The papillomavirus E7 proteins. Virology.

[33] Wang JW, Roden RB (2013). Virus-like particles for the prevention of human papillomavirus-associated malignancies. Expert Rev. Vaccines.

[34] Tumban E, Peabody J, Tyler M, Peabody DS, Chackerian B (2012). VLPs Displaying a Single L2 Epitope Induce Broadly Cross-Neutralizing Antibodies against Human Papillomavirus. PLoS ONE.

[35] Buck CB, Trus BL (2012). The papillomavirus virion: a machine built to hide molecular Achilles' heels. Adv. Exp. Med. Biol..

[36] Negahdaripour M (2017). A novel HPV prophylactic peptide vaccine, designed by immunoinformatics and structural vaccinology approaches. Infection, Genet. Evolut.: J. Mol. Epidemiol. Evol. Genet. Infectious Dis..

[37] Negahdaripour M (2018). Structural vaccinology considerations for in silico designing of a multi-epitope vaccine. Infection, Genet. Evolut: J. Mol. Epidemiol. Evolut. Genet. Infect. Dis..

[38] Sanami S (2021). Design of a multi-epitope vaccine against cervical cancer using immunoinformatics approaches. Sci. Rep..

[39] DiMaio D, Mattoon D (2001). Mechanisms of cell transformation by papillomavirus E5 proteins. Oncogene.

[40] Straight SW, Herman B, McCance DJ (1995). The E5 oncoprotein of human papillomavirus type 16 inhibits the acidification of endosomes in human keratinocytes. J. Virol..

[41] Leechanachai P, Banks L, Moreau F, Matlashewski G (1992). The E5 gene from human papillomavirus type 16 is an oncogene which enhances growth factor-mediated signal transduction to the nucleus. Oncogene.

[42] Regan JA, Laimins LA (2008). Bap31 is a novel target of the human papillomavirus E5 protein. J. Virol..

[43] Cortese MS, Ashrafi GH, Campo MS (2010). All 4 di-leucine motifs in the first hydrophobic domain of the E5 oncoprotein of human papillomavirus type 16 are essential for surface MHC class I downregulation activity and E5 endomembrane localization. Int. J. Cancer.

[44] Namvar A, Panahi HA, Agi E, Bolhassani A (2020). Development of HPV16,18,31,45 E5 and E7 peptides-based vaccines predicted by immunoinformatics tools. Biotech. Lett..

[45] Mizuuchi M (2012). Novel oligomannose liposome-DNA complex DNA vaccination efficiently evokes anti-HPV E6 and E7 CTL responses. Exp. Mol. Pathol..

[46] Bourgault Villada I (2000). Identification in humans of HPV-16 E6 and E7 protein epitopes recognized by cytolytic T lymphocytes in association with HLA-B18 and determination of the HLA-B18-specific binding motif. Eur. J. Immunol..

[47] Matijevic M (2011). Immunization with a poly (lactide co-glycolide) encapsulated plasmid DNA expressing antigenic regions of HPV 16 and 18 results in an increase in the precursor frequency of T cells that respond to epitopes from HPV 16, 18, 6 and 11. Cell. Immunol..

[48] Ressing ME (1995). Human CTL epitopes encoded by human papillomavirus type 16 E6 and E7 identified through in vivo and in vitro immunogenicity studies of HLA-A*0201-binding peptides. J. Immunol..

[49] Rudolf MP, Fausch SC, Da Silva DM, Kast WM (2001). Human dendritic cells are activated by chimeric human papillomavirus type-16 virus-like particles and induce epitope-specific human T cell responses in vitro. J. Immunol..

[50] Feltkamp MC (1993). Vaccination with cytotoxic T lymphocyte epitope-containing peptide protects against a tumor induced by human papillomavirus type 16-transformed cells. Eur. J. Immunol..

[51] Doytchinova IA, Flower DR (2007). VaxiJen: a server for prediction of protective antigens, tumour antigens and subunit vaccines. BMC Bioinformatics.

[52] Dhanda SK, Vir P, Raghava GP (2013). Designing of interferon-gamma inducing MHC class-II binders. Biol. Direct.

[53] Mohan T, Mitra D, Rao DN (2014). Nasal delivery of PLG microparticle encapsulated defensin peptides adjuvanted gp41 antigen confers strong and long-lasting immunoprotective response against HIV-1. Immunol. Res..

[54] Saha S, Raghava GP (2006). Prediction of continuous B-cell epitopes in an antigen using recurrent neural network. Proteins.

[55] Roy A, Kucukural A, Zhang Y (2010). I-TASSER: a unified platform for automated protein structure and function prediction. Nat. Protoc..

[56] Pettersen EF (2004). UCSF Chimera–a visualization system for exploratory research and analysis. J. Comput. Chem..

[57] Humphrey W, Dalke A, Schulten K (1996). VMD: Visual molecular dynamics. J. Mol. Graph..

[58] Phillips JC (2005). Scalable molecular dynamics with NAMD. J. Comput. Chem..

[59] Grote A (2005). JCat: A novel tool to adapt codon usage of a target gene to its potential expression host. Nucleic Acids Res..

[60] Abraham Peele K, Srihansa T, Krupanidhi S, Ayyagari VS, Venkateswarulu TC (2021). Design of multi-epitope vaccine candidate against SARS-CoV-2: A in-silico study. J. Biomol. Struct. Dyn..

[61] Rapin N, Lund O, Bernaschi M, Castiglione F (2010). Computational immunology meets bioinformatics: the use of prediction tools for molecular binding in the simulation of the immune system. PLoS ONE.

